# Associations Between County and Municipality Zoning Ordinances and Access to Fruit And Vegetable Outlets in Rural North Carolina, 2012

**DOI:** 10.5888/pcd10.130196

**Published:** 2013-12-05

**Authors:** Mariel Leah Mayo, Stephanie B. Jilcott Pitts, Jamie F. Chriqui

**Affiliations:** Author Affiliations: Mariel Leah Mayo, Albemarle Regional Health Services–North Carolina, Elizabeth City, North Carolina; Jamie F. Chriqui, University of Illinois at Chicago, Chicago, Illinois. At the time of the study, Ms Mayo was affiliated with East Carolina University, Greenville, North Carolina.

## Abstract

**Introduction:**

Zoning ordinances and land-use plans may influence the community food environment by determining placement and access to food outlets, which subsequently support or hinder residents’ attempts to eat healthfully. The objective of this study was to examine associations between healthful food zoning scores as derived from information on local zoning ordinances, county demographics, and residents’ access to fruit and vegetable outlets in rural northeastern North Carolina.

**Methods:**

From November 2012 through March 2013, county and municipality zoning ordinances were identified and double-coded by using the Bridging the Gap food code/policy audit form. A healthful food zoning score was derived by assigning points for the allowed use of fruit and vegetable outlets. Pearson coefficients were calculated to examine correlations between the healthful food zoning score, county demographics, and the number of fruit and vegetable outlets. In March and April 2013, qualitative interviews were conducted among county and municipal staff members knowledgeable about local zoning and planning to ascertain implementation and enforcement of zoning to support fruit and vegetable outlets.

**Results:**

We found a strong positive correlation between healthful food zoning scores and the number of fruit and vegetable outlets in 13 northeastern North Carolina counties (*r* = 0.66, *P* = .01). Major themes in implementation and enforcement of zoning to support fruit and vegetable outlets included strict enforcement versus lack of enforcement of zoning regulations.

**Conclusion:**

Increasing the range of permitted uses in zoning districts to include fruit and vegetable outlets may increase access to healthful fruit and vegetable outlets in rural communities.

## Introduction

Obesity is a growing public health problem, particularly in the rural United States (1–3). The increased rates of obesity in the rural US may be partially related to the community food environment (4–7) or the distribution of food outlets within a community (8). The food environment may influence residents’ dietary behaviors and thus may be associated with obesity (8–12). Fewer healthful food outlets and more convenience stores and fast food restaurants are associated with increased rates of obesity (5,11,12).

For the purposes of this article, fruit and vegetable outlets refer to farmers markets, roadside stands, fruit and vegetable markets, and fruit and vegetable carts. Fruits and vegetables are more often available at these outlets than at convenience stores and fast food restaurants (13), and thus, fruit and vegetable outlets may increase access to and consumption of fruits and vegetables (9,14,15). A diet rich in fruits and vegetables is associated with lower body mass index (16). Thus, the promotion and enhancement of fruit and vegetable outlets in rural, low-income communities could be a way to increase consumption of fruits and vegetables and decrease the prevalence and incidence of obesity among residents.

In rural and low-income communities, land-use policies may hinder the development of fruit and vegetable outlets. A recent study showed that local zoning ordinances are less likely to allow farmers markets as a permitted use of land than they are to allow other food outlets (eg, supermarkets, convenience stores) (17). The same study found that low-income communities were less likely than high-income communities to zone for farmers markets as a permitted land use (17). Although zoning ordinances and land-use policies have the potential to support or hinder the development of fruit and vegetable outlets, to our knowledge no studies have examined the relationship between zoning ordinances and access to fruit and vegetable outlets. The objective of this study was to examine associations between healthful food zoning scores as derived from local zoning ordinances, county demographics, and access to fruit and vegetable outlets in rural northeastern North Carolina. An additional objective was to qualitatively examine perspectives among knowledgeable municipal planners on implementation and enforcement of zoning to support fruit and vegetable outlets.

## Methods

### The North Carolina Community Transformation Grant Program

In 2011, the North Carolina Division of Public Health was awarded more than $7.4 million per year for 5 years from the Centers for Disease Control and Prevention through the Community Transformation Grant (CTG) Program to pursue 4 ways of improving health and reducing health-related disparities: 1) tobacco-free living, 2) active living, 3) healthful eating, and 4) high-impact, evidence-based clinical and other preventive services (18). In North Carolina, a portion of this funding was allocated to increase the number of communities that support fruit and vegetable outlets.

### Study setting

The CTG Project Region 9 Collaborative includes 15 counties in rural northeastern North Carolina: Bertie, Camden, Chowan, Currituck, Dare, Edgecombe, Gates, Hertford, Hyde, Martin, Northampton, Pasquotank, Perquimans, Tyrell, and Washington (Table 1). Region 9 counties have high rates of obesity and poverty; an average 33.8% of the adult population is obese, compared with 30.7% of the adult population in North Carolina and 30.3% of the adult population in the United States (2). The poverty rate is 20.5%, exceeding the rate for North Carolina (17.4%) and the United States (15.4%) (20). Region 9 counties are predominantly rural; an average of 77.9% of residents live in rural areas, and in 3 counties, all residents live in rural areas (22). The mean percentage of African American residents in Region 9 is 37% (range, 2.8%–62.9%) (19).

**Table 1:** Demographic Characteristics of the Community Transformation Grant Project Region 9 Counties, North Carolina

County	Non-Hispanic African American^a^, %	Non-Hispanic White^a^, %	Poverty Estimates^b^, %	Population Living in Food Desert^c^, %	Obesity Rates, 2008–2010^d^	Population Rural^e^, %
**Bertie**	62.9	34.9	27.0	6.6	37.5	83.2
**Camden**	13.6	82.4	9.7	0	32.3	99.6
**Chowan**	34.6	61.6	21.1	7.7	30.1	67.6
**Currituck**	6.0	89.7	11.1	0	32.6	98.3
**Dare**	2.8	89.7	12.3	0	28.6	29.0
**Edgecombe**	57.7	38.0	24.5	10.4	40.2	45.3
**Gates**	34.1	63.7	17.5	0	34.8	100.0
**Hertford**	60.9	34.7	26.1	29.5	35.4	68.6
**Hyde**	32.4	59.9	21.9	16.6	32.2	100.0
**Martin**	43.8	52.5	23.4	0	35.5	78.1
**Northampton**	58.8	39.2	22.5	0	34.2	89.4
**Pasquotank**	38.5	55.8	22.9	17.6	33.3	41.3
**Perquimans**	25.2	72.1	16.4	0	33.4	100.0
**Tyrrell**	38.8	53.8	28.7	0	31.9	100.0
**Washington**	50.1	45.8	22.4	13.0	34.6	67.8
**Regional average**	37.0	58.2	20.5	6.8	33.8	77.9
**North Carolina**	21.5	68.5	17.4	NA	27.8	33.9

Abbreviation: NA, not applicable.

a Source: US Department of Commerce (19).

b Source: US Department of Commerce (20).

c Source: US Department of Agriculture, Economic Research Service (21).

d Source: Centers for Disease Control and Prevention, National Diabetes Surveillance System (2).

e Source: US Department of Commerce, 2010 Census Urban and Rural Classification and Urban Area Criteria (22).

Because they lacked zoning ordinances, Hyde and Tyrrell counties were excluded from our study. On the basis of the definition of *rural* used by the Office of Management and Budget, all Region 9 counties are rural, with the exception of Currituck and Edgecombe (23). In 2011, 10 of the 15 counties were identified by the North Carolina Department of Commerce as being among the 40 most economically distressed counties of the state’s 100 counties (24). 

### Zoning ordinances

The Bridging the Gap Community Obesity Measures Project (BTG-COMP) developed policy coding forms to examine the relationship between the built environment and health and local government laws and policies (25). Between November 2012 and March 2013, the study team used the BTG-COMP food code/policy audit form (audit form) — which was also used in a prior study (17) — to code and score each CTG Project Region 9 county and municipality zoning ordinance. In this article, “municipality” refers to cities and towns. The most recent versions of zoning ordinances were found through Internet searches, starting with each county’s website. County and municipality planners were contacted for paper copies of ordinances not found online. Sections of the audit form were completed according to the BTG coding protocol (25), with 5 modifications to the protocol to address the rural nature of the study setting. For example, 1 section of the audit form is on “urban agriculture.” We changed this term to “rural agriculture,” which was coded according to any term that included horticultural or agricultural uses, farming, farms, community gardens, or the sale of produce on the property where grown. This term also included voluntary agricultural districts for farmland or exempt bona fide farms. The ordinances were independently coded by 2 coders, who then met to discuss and resolve discrepancies. Initial interrater reliability ranged from 65% to 100% (mean, 88.9%). All disagreements in coding were resolved through consensus until the final interrater reliability was 100%.

### Healthful food zoning score

Each county or municipality was assigned a healthful food zoning score related to the elements of the zoning ordinance supportive of healthful food outlets. The coder first ascertained whether any of 7 possible zoning districts were included in the ordinance: agricultural, code reform, commercial, mixed use, public/civic/government/school, recreation/open space, and residential. The audit form also has 6 subsections for the following types of food outlets: farmers markets, green/fresh fruit and vegetable carts, mobile food vendors/carts, urban agriculture, produce/fresh fruit and vegetable stands, and produce/fruit market/stores. The coder then reviewed each of the 6 food outlet subsections in each district, giving the municipality 1 point if the topic of food outlets was addressed in any way. If the topic was addressed, points were assigned according to the type of uses that were allowed in that district: The municipality was assigned 4 points if the use was permitted, 3 points if conditional, 2 points if accessory, 1 point if prohibited, and 0 points if type of use was not specified.

After the zoning ordinances were coded, the healthful food zoning score for each county and municipality was calculated as the total number of points assigned to its ordinance, divided by the highest possible number of points that could be assigned based on the number of zoned districts. The county healthful food zoning score was the weighted average of the municipality healthful food zoning scores, derived by multiplying the healthful food zoning scores by the proportion of the total county population residing in the municipalities, and then summing these scores. For example, if county *x* has only 1 municipality (municipality *y*), if 20% of county residents live in municipality *y*, and if the healthful food zoning score for county *x* is 0.25 and for municipality *y* is 0.40, then the weighted healthful food zoning score was calculated as (0.20 × 0.40) + (0.80 × 0.25) = 0.28. When we found only a county zoning ordinance (no municipality zoning ordinance), we assigned a weight of 1 to the county healthful food zoning score. Possible healthful food zoning scores ranged from 0 to 1, with a higher score indicating healthier food zoning.

### North Carolina Fruit and Vegetable Non-Retail Outlet Inventory

Access to fruit and vegetable outlets was quantified by using data from the state-developed North Carolina Fruit and Vegetable Non-Retail Outlet Inventory. From June through December 2012, the 2012 inventory was used to catalog fruit and vegetable outlets. Data for Region 9 outlets were found through an Internet search; the Internet search included but was not limited to searches for farmers markets, roadside stands, farm stands, fruit and vegetable outlets, and tailgate markets. Outlet locations were verified through telephone calls and e-mails with local Cooperative Extension agencies, on-site verification, active Facebook accounts, Facebook communication, e-mail correspondence, and face-to-face and telephone interviews with market owners, managers, and employees. The number of fruit and vegetable outlets per county ranged from 1 to 10.

### Quantitative data analysis

Pearson correlation coefficients were used to examine the correlations between the supportiveness of zoning ordinances for fruit and vegetable outlets as measured by the healthful food zoning score, number of fruit and vegetable outlets per county, and county sociodemographic characteristics. We used SAS 9.3 (SAS Institute Inc, Cary, North Carolina) for analyses and an α less than .05 to determine significance. Cohen’s criteria were used to determine the strength of the correlations (weak correlations, 0–0.29; moderate correlations, 0.30–0.49; and strong correlations, ≥0.50) (26).

### Planner qualitative interviews

The purpose of the qualitative interview was to gain an understanding of the level of implementation and enforcement of zoning ordinances. A list of contacts knowledgeable about local zoning was generated from staff listings on county and municipality websites and the North Carolina League of Municipalities directory. The contacts were typically the county or municipality planner or planning director. Planners were contacted via e-mail and asked about their willingness to complete a telephone interview on local zoning. No identifying information was collected during the telephone interview, and participation was voluntary. The planner interview study protocol was reviewed and approved by the institutional review board of East Carolina University. Planners from all 28 counties were contacted for qualitative interviews, and 9 planners from 5 counties agreed to be interviewed. A qualitative interview guide was adapted from the Community Planning Survey from the Department of City and Regional Planning (University of North Carolina at Chapel Hill) (27). The interview guide included open- and close-ended questions on how zoning ordinances were implemented and enforced. For example, “To what extent do local zoning ordinances and land use laws and policies support farmers markets and stands?”

### Qualitative data analysis

Using the qualitative interview guide, a study team member conducted telephone interviews of planners. Detailed notes were taken during the interview and were reviewed immediately after the call to add any information that may have been missed. An inductive approach was used by reading the notes several times and looking for similarities and differences. From this review, a codebook was used to further organize responses into themes. The codebook included codes such as “permit needed,” “town supportive,” and “zoning prohibits.” Responses for each question were reviewed separately and coded accordingly.

## Results

The regional average of the unweighted healthful food zoning scores in the 11 counties and 17 municipalities was 0.33 (standard deviation [SD], 0.21; range 0–0.83). The mean number of fruit and vegetable outlets for the region was 3.5 (SD, 2.7) (Table 2). We found a strong, positive, significant correlation between healthful food zoning scores and number of fruit and vegetable outlets (*r* = 0.66, *P* = .01) (Table 3). We found a moderate inverse (but not significant) correlation between healthful food zoning scores and the percentage of African American county residents (*r* = −0.47, *P* = .11) and a moderate positive (but not significant) correlation between healthful food zoning scores and the percentage of white residents (*r* = 0.48, *P* = .10). We also found a moderate inverse (but not significant) correlation between the healthful food zoning scores and percentage of county residents living in poverty (*r *= −0.47, *P* = .11).

**Table 2:** Number of Healthful Fruit and Vegetable Outlets and Healthful Food Zoning Scores for the Community Transformation Grant Project Region 9 Counties, North Carolina

County	No. of Fruit and Vegetable Outlets^a^	Healthful Food Zoning Score (Weighted)^b^
Bertie	1	0.09
Camden	2	0.40
Chowan	4	0.64
Currituck	10	0.83
Dare	5	0.17
Edgecombe	4	0.44
Gates	1	0.36
Hertford	2	0.14
Hyde	3	—c
Martin	2	0.06
Northampton	1	0.18
Pasquotank	5	0.36
Perquimans	7	0.32
Tyrrell	1	—c
Washington	2	0.33
Regional average per county	3.5	NC
North Carolina average per county	6.9	NC

Abbreviation: NC, not calculated.

a Source: Community Transformation Grant Project 2012, North Carolina Fruit and Vegetable Non-Retail Outlet Inventory.

b Weighted healthful food zoning scores derived for each county by multiplying county and/or municipality unweighted healthful zoning scores by population proportion and then summing. Possible healthful food zoning scores ranged from 0 to 1, with a higher score indicating healthier food zoning.

c Counties were excluded from our analysis because they lacked zoning ordinances.

**Table 3:** Bivariate Associations Between Healthful Food Zoning Scores and Number of Fruit and Vegetable Outlets and County-Level Sociodemographic Characteristics in 13 Counties in North Carolina

Characteristic	Healthful Food Zoning Score	
	Pearson Correlation, *r*	*P* Value
No. of fruit and vegetable outlets	0.66	.01
Percentage African American	−0.47	.10
Percentage white	0.48	.10
Obesity rates	−0.25	.40
Percentage living in poverty	−0.47	.11
Percentage living in food desert	−0.13	.66
Percentage living in a rural area	0.17	.58

Two themes emerged from analysis of the qualitative interviews: municipalities that had strict enforcement of zoning regulations and counties and municipalities that lacked enforcement. All participants were receptive to the idea of adding healthful food–related language into zoning ordinances.

Four of the 9 planners, all of whom were municipality planners, spoke of the strict enforcement of zoning rules on development of a fruit or vegetable outlet in their municipalities. These municipalities were generally located on the coast and were more urban and had more tourism than the other Region 9 municipalities. Municipalities of this type would not allow the operation of a fruit or vegetable outlet on land not zoned for such uses. Furthermore, permits were required to open a fruit and vegetable outlet. For example, Participant 7 stated, “We don’t allow them [farmers markets/produce stands] right now, so for them to be allowed, someone would have to come in with a request, and the planning board and town council would have to review the application.”

Five planners reported a lack of enforcement of zoning regulations when a produce stand opens, unless there is a complaint. Counties and municipalities of this type were more rural than other Region 9 counties. Participant 1 stated, “We are very lax because we are a very rural county and farming is still king around here . . . 98.2% of our county is zoned AR-30 (rural residential) . . . and in AR-30 zoning districts those type farmers markets and produce stands are allowed by right . . . and also we don’t try to govern them very much. . . . We actually try to support them as best we can.” Participant 2 reported, “No property in town is zoned for these [farmers market/produce stand] uses, but since they are temporary no one enforces the zoning regulations.”

## Discussion

Our study found a significant positive correlation between the healthful food zoning score and the number of fruit and vegetable outlets. Furthermore, the mean unweighted healthful food zoning score for the 13 counties in our study was low (0.33 on a scale from 0 to 1). The low healthful food zoning scores are in agreement with results of a previous study, which indicated that zoning ordinances are less likely to address and permit fruit and vegetable outlets than they are to address and permit other food outlets (17).

Our findings indicate that addressing healthful food outlets in zoning ordinances may translate into increased access to such outlets. Zoning ordinances that do not specifically allow healthful food outlets may hinder the development of farmers markets (15). For instance, allowing farmers markets as a land use in several zoning districts (eg, commercial, mixed-use, residential) may translate into greater access to healthful food outlets. On the other hand, our qualitative data indicate that in some rural areas, such as those where “farming is king,” a lack of enforcement of zoning for fruit and vegetable outlets enables their establishment. Thus, allowing healthful food outlets may be important only in municipalities where zoning ordinances are strictly enforced. Qualitative interview data provided a deeper understanding of how zoning ordinances are enforced in the counties studied and how important the ordinances are in determining location of a fruit and vegetable outlet.

Our study has several limitations. One, although independent coders reached consensus on coding for each ordinance, the coding of ordinances was subjective. Two, our study was an ecological analysis of county-level data and would have been improved by including individual-level data. Three, although detailed notes were taken, the planner interviews were not audio-recorded, and data were not analyzed using qualitative software. Four, the rural setting of our study may have created difficulty in obtaining data on zoning ordinances for all counties and municipalities of interest. This last limitation probably led to the small sample size of planners who completed the qualitative interviews. 

We also note several strengths of our study. One, zoning ordinances were codified before data on fruit and vegetable outlets were collected; this codification is necessary because it takes time for policies to be implemented and translated into environmental changes (eg, more healthful food outlets). Two, although the sample size for the planner interviews was small (n = 9), the planners represented various viewpoints and perspectives from 5 counties. Three, we used qualitative interviews coupled with quantitative data; the qualitative interviews provided in-depth information on zoning and land-use policies that could not be obtained through the healthful food zoning scores alone. Additional strengths of this study included the use of 2 independent coders to reduce any bias that may have resulted from using only 1 coder, the use of a standardized coding protocol, and discussion between 2 independent coders to ensure coding reliability.

Our study offers a potential evaluation approach for the CTG Project strategy to increase health-related language in zoning and land-use plans. Our study data can be used as baseline data to assess improvements at the conclusion of the CTG Project (18). The approach and findings presented here provide a guide for beginning partnerships between public health and planning professionals. Our approach can also serve as an assessment for public health or planning officials to determine areas for improvement in their zoning regulations and land-use plans. The *CDC Guide to Strategies to Increase the Consumption of Fruits and Vegetables* supports the analysis of existing zoning ordinances and land-use policies to identify barriers to the development of fruit and vegetable outlets (15). The guide suggests working with local planners to modify regulations and policies to support the establishment of fruit and vegetable outlets (15). For projects that encourage alterations to zoning ordinances to include healthful food outlets as allowable land use, the approach used in this study can demonstrate whether such zoning alterations translate into greater access to healthful food outlets, ultimately improving the community food environment.

## References

[1] Flegal KM , Carroll MD , Ogden CL , Curtin LR . Prevalence and trends in obesity among US adults, 1999–2008. JAMA 2010;303(3):235–41. 10.1001/jama.2009.2014 20071471

[2] Centers for Disease Control and Prevention. County-level estimates of obesity — state maps (2009). Diabetes data and trends. http://apps.nccd.cdc.gov/DDT_STRS2/CountyPrevalenceData.aspx?mode=OBS. Accessed February 15, 2013.

[3] Patterson PD , Moore CG , Probst JC , Shinogle JA . Obesity and physical inactivity in rural America. J Rural Health 2004;20(2):151–9. 10.1111/j.1748-0361.2004.tb00022.x 15085629

[4] Hill JO , Peters JC . Environmental contributions to the obesity epidemic. Science 1998;280(5368):1371–4. 10.1126/science.280.5368.1371 9603719

[5] Jilcott SB , Wade S , McGuirt JT , Wu Q , Lazorick S , Moore JB . The association between the food environment and weight status among eastern North Carolina youth. Public Health Nutr 2011;14(9):1610–7. 10.1017/S1368980011000668 21486525

[6] Rundle A , Neckerman KM , Freeman L , Lovasi GS , Purciel M , Quinn J , Neighborhood food environment and walkability predict obesity in New York City. Environ Health Perspect 2009;117(3):442–7. 19337520 10.1289/ehp.11590 PMC2661915

[7] Leung CW , Laraia BA , Kelly M , Nickleach D , Adler NE , Kushi LH , The influence of neighborhood food stores on change in young girls’ body mass index. Am J Prev Med 2011;41(1):43–51. 10.1016/j.amepre.2011.03.013 21665062 PMC3115539

[8] Glanz K , Sallis JF , Saelens BE , Frank LD . Healthy nutrition environments: concepts and measures. Am J Health Promot 2005;19(5):330–3. 10.4278/0890-1171-19.5.330 15895534

[9] Evans AE , Jennings R , Smiley AW , Medina JL , Sharma SV , Rutledge R , Introduction of farm stands in low-income communities increases fruit and vegetable among community residents. Health Place 2012;18(5):1137–43. 10.1016/j.healthplace.2012.04.007 22608130

[10] Morland KB , Evenson KR . Obesity prevalence and the local food environment. Health Place 2009;15(2):491–5. 10.1016/j.healthplace.2008.09.004 19022700 PMC4964264

[11] Morland K , Diez Roux AV , Wing S . Supermarkets, other food stores, and obesity: the Atherosclerosis Risk in Communities study. Am J Prev Med 2006;30(4):333–9. 10.1016/j.amepre.2005.11.003 16530621

[12] Jilcott SB , Keyserling T , Crawford T , McGuirt JT , Ammerman AS . Examining associations among obesity and per capita farmers’ markets, grocery stores/supermarkets, and supercenters in US counties. J Am Diet Assoc 2011;111(4):567–72. 10.1016/j.jada.2011.01.010 21443990

[13] Morland K , Filomena S . Disparities in the availability of fruits and vegetables between racially segregated urban neighbourhoods. Public Health Nutr 2007;10(12):1481–9. 10.1017/S1368980007000079 17582241

[14] Ruelas V , Iverson E , Kiekel P , Peters A . The role of farmers’ markets in two low income, urban communities. J Community Health 2012;37(3):554–62. 10.1007/s10900-011-9479-y 21922162

[15] The CDC guide to strategies to increase the consumption of fruits and vegetables. http://www.cdc.gov/obesity/downloads/FandV_2011_WEB_TAG508.pdf. Accessed September 12, 2012.

[16] Azagba S , Sharaf MF . Fruit and vegetable consumption and body mass index: a quantile regression approach. J Prim Care Community Health 2012;3(3):210–20. 10.1177/2150131911434206 23803782

[17] Chriqui JF , Thrun E , Rimkus L , Barker DC , Chaloupka FJ . Zoning for healthy food access varies by community income — a BTG research brief. Chicago (IL): Bridging the Gap Program, Health Policy Center, Institute for Health Research and Policy, University of Illinois at Chicago; 2012.

[18] Centers for Disease Control and Prevention. Community Transformation Grants (CTG) states and communities programs description. http://www.cdc.gov/communitytransformation/funds/programs.htm. Accessed May 22, 2012.

[19] United States Department of Commerce. State and county quick facts, 2010. http://quickfacts.census.gov/qfd/states/37/37015.html. Accessed June 1, 2013.

[20] United States Department of Commerce. Small area income and poverty estimates. US and all state and counties [XLS]. State and county estimates for 2010. http://www.census.gov/did/www/saipe/data/statecounty/data/2010.html. Updated December 12, 2012. Accessed February 14, 2013.

[21] US Department of Agriculture Economic Research Service. Food desert locator: documentation. http://www.ers.usda.gov/data-products/food-desert-locator/documentation.aspx. Accessed November 21, 2012.

[22] United States Department of Commerce. Percent urban and rural in 2010 by state and county [XLS]. 2010 Census urban and rural classification and urban area criteria. http://www.census.gov/geo/reference/urban-rural-2010.html. Updated January 30, 2013. Accessed February 14, 2013.

[23] US Department of Commerce Economic and Statistical Administration. US Census Bureau. North Carolina — core based statistical areas, counties, and independent cities, November 2004. http://www2.census.gov/geo/maps/metroarea/stcbsa_pg/Nov2004/cbsa2004_NC.pdf.

[24] North Carolina Department of Commerce. 2011 County tier designations. http://www.nccommerce.com/research-publications/incentive-reports/2011-county-tier-designations. Accessed May 23, 2012.

[25] Bridging the Gap Community Obesity Measures Project: policy coding protocol. Chicago (IL): University of Illinois at Chicago, Institute for Health Research. Updated March 23, 2012.

[26] Cohen J . Statistical power analysis for the behavioral sciences, 2nd edition. Hillsdale (NJ): Lawrence Erlbaum Associates; 1988.

[27] Evenson KR , Aytur SA , Satinsky SB , Rodriguez DA . Barriers to municipal planning for pedestrians and bicyclists in North Carolina. N C Med J 2011;72(2):89–97. 21721492

